# Influence of Internal Thoracic Artery Harvesting on Sternal Osteoblastic Activity and Perfusion

**DOI:** 10.3390/diagnostics10110921

**Published:** 2020-11-09

**Authors:** Sergey Mamchur, Yuri Vecherskii, Tatiana Chichkova

**Affiliations:** 1 Department of Cardiovascular Surgery, Research Institute for Complex Issues of Cardiovascular Diseases, 650002 Kemerovo, Russia; chi4cova@yandex.ru; 2 Department of Cardiovascular Surgery, Research Institute of Cardiology, 634012 Tomsk, Russia; yvsurgeon@gmail.com

**Keywords:** sternal perfusion, coronary artery bypass graft, internal thoracic artery, single-photon emission computed tomography

## Abstract

The purpose of this study was to assess the sternal osteoblastic activity and perfusion in the early period after a coronary artery bypass graft (CABG) using single-photon emission computed tomography (SPECT) and three-phasic dynamic scintigraphy (3PDS) with 99mTc methylene diphosphonate (MDP). Methods: The study group consisted of 57 male patients that were 57.3 ± 6.6 years of age. Thirty-six of them were randomized into two groups: in group I (*n* = 18), the internal thoracic artery (ITA) was skeletonized, and in group II (*n* = 18), the ITA was pedicled. All the patients in groups I and II underwent an off-pump CABG using 1.7 ± 0.3 grafts, including one anastomosis of the ITA to the left anterior descending coronary artery. The control group III (*n* = 21) consisted of patients that underwent mitral valve repair surgery, in whom the sternotomy without the ITA harvesting was performed. The 3PDS and SPECT of the thorax with 99mTc MDP were performed 2 weeks after surgery. Results: The sternal phosphates uptake in group I was approximately twice as high as in group II and approximately 1.5 times higher than in group III (*p* < 0.05). The MDP uptake asymmetry after the ITA skeletonization was the same as in the group with both intact ITAs. In contrast, after the pedicled ITA harvesting, the osteoblastic activity of the ipsilateral side of the sternum was lower than in the contralateral one. There was no statistically significant difference in scintillation count in the xiphoid process between groups I and II (*p* > 0.05); however, we observed a significant difference in the manubrium and body (*p* < 0.05). Conclusion: The main factor that improved the sternal perfusion after a CABG was the preservation of branches supplying the sternum using the skeletonization technique of ITA harvesting.

## 1. Introduction

A high incidence of postoperative sternal infections results in an increase in hospital stay, mortality, and treatment costs [1,2,3], and causes skepticism among cardiac surgeons about the use of both internal thoracic arteries (ITA) for a coronary artery bypass graft (CABG). Several risk factors are proven to be the reasons for postoperative sternal perfusion deterioration. One of the most important risk factors is bilateral ITA harvesting [4,5,6], which seems to lead to sternal perfusion worsening [7] or sternal injury during ITA harvesting [5].

Few data are available about the decrease of ischemic sternal osteomyelitis incidence after ITA skeletonization, even the bilateral one [8,9]. The theoretical background of the anti-ischemic effect of skeletonization is that this harvesting technique preserves anatomically intact sternal branches of the anterior intercostal arteries, which originate from the ITA. In the pedicled harvesting technique, these branches are transected and the sternum loses its main blood supply source [10]. The study of the osteoblastic activity and reparation of the sternum after ITA harvesting is highly important because it can give information regarding the risk assessment of postoperative sternal infections.

The three types of vessels supplying the sternum are identified as follows [11]: (a) sternal performant branches of the ITA supplying the sternum and pectoralis major muscle; (b) sternal intercostal branches of the ITA supplying the sternum and intercostal spaces; (c) posterior intercostal arteries, which have no anastomoses with the ITA but intersect it on their way to the sternum. All three types of these arteries are located mostly in the proximal part of the sternum. This is important for a correct interpretation of sternal scintigraphy after a total or partial sternotomy. A local decrease of phosphates uptake can identify the compromised vessel. Up to date, there have been no studies of sternal perfusion using three-phasic dynamic scintigraphy (3PDS).

The purpose of this study was to assess the sternal osteoblastic activity and perfusion in the early period after a CABG using single-photon emission computed tomography (SPECT) and 3PDS with 99mTc methylene diphosphonate (MDP).

## 2. Materials and Methods

The study was approved by the local ethics committee. Informed consent was obtained from all the patients. The study group consisted of 57 male patients that were 57.3 ± 6.6 years of age and did not have diabetes mellitus or chronic obstructive pulmonary disease (COPD). Thirty-six of them were randomized into two groups: in group I (*n* = 18), the ITA was skeletonized, and in group II (*n* = 18), the ITA was pedicled. All patients of groups I and II underwent an off-pump CABG using 1.7 ± 0.3 grafts, including one anastomosis of the ITA to left anterior descending (LAD) coronary artery. The control group III (*n* = 21) consisted of patients that underwent mitral valve repair surgery, in whom the sternotomy without ITA harvesting was performed. In all cases, sternal closure was performed via anchoring with steel wires. This closure method is cost-effective and has low rates of sternal wound complications. The clinical characteristics of the patients are presented in Table 1.

**Figure 1:**
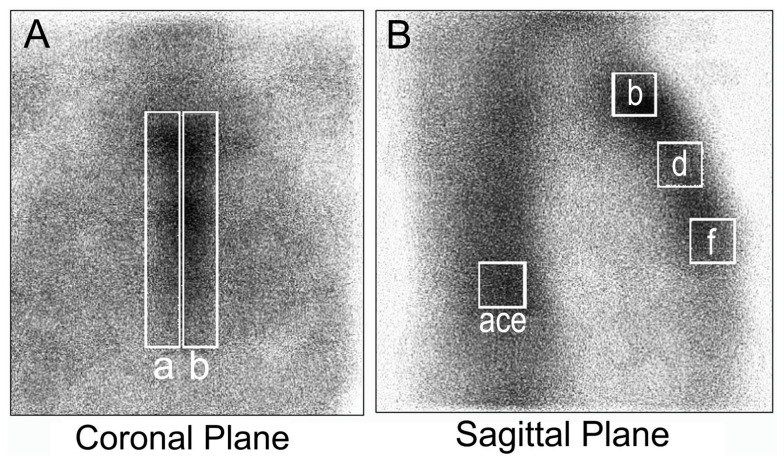
The regions of interest (ROIs) for relative methylene diphosphonate (MDP) uptake in the coronal (contralateral to internal thoracic artery (ITA) harvesting “a” and ipsilateral “b” areas) plane (**A**) and the sagittal plane (**B**): a, c, and e areas of the vertebral column, and b, d, and f areas of sternal manubrium, body, and xiphoid process, respectively.

3PDS and SPECT of the thorax with 99mTc MDP were performed 2 weeks after surgery using a tomographic gamma scanner Omega 500 (TechniCare, Solon, OH, USA). After an intravenous injection of 5 MBq/kg of 99mTc MDP (Technephor, Diamed, Moscow, Russia), the 3PDS was immediately performed in the sagittal plane using a low-energy, high-resolution collimator with a 140 keV photopeak energy. SPECT was performed 3 h later [12]. The emission was registered during a detector rotation around a longitudinal axis of a body in 32 planes for 30 s each. Tomographic sections with a two-voxel thickness were reconstructed in the sagittal and frontal planes. A specialist in nuclear medicine that was blinded to the patients’ group performed the quantitative analysis. In the sagittal plane, the scintillation rate was counted in manually drawn rectangular regions of interest (ROIs) corresponding to the manubrium, body, and xiphoid process of the sternum. The MDP uptake was expressed in percent in comparison to a vertebral column, the scintillation count of which was conditionally taken as 100%. In the coronal plane, the MDP uptake was calculated ipsilaterally to the ITA harvesting side of the sternum in comparison to the opposite side (Figure 1).

The variables are presented as absolute values and a percentage of the overall group quantity, or median and quartile range. The statistical analysis was performed in MedCalc v. 17.2 (source Medcalc Software, Ostend, Belgium) using chi-square, Dunn’s, and median tests. A *p*-value < 0.05 was considered statistically significant.

## 3. Results

### 3.1. The Osteoblastic Activity of the Sternum after the ITA Harvesting, as Estimated Using SPECT

The results of the relative MDP uptake presented in Table 2 demonstrate that the sternal osteoblastic activity in group I (skeletonized ITA) was higher than in the other groups.

Table 2 demonstrates that the sternal phosphates uptake in group I was approximately twice as high as in group II and approximately 1.5 times higher than in group III (*p* < 0.05). This means that the osteoblastic activity after the pedicled ITA harvesting was lower than after a sternotomy without the ITA harvesting, but after the ITA skeletonization, the sternal osteoblastic activity was even higher than after a sternotomy with both intact ITAs. The MDP uptake asymmetry after the ITA skeletonization was the same as in the group with both intact ITAs. In contrast, after the pedicled ITA harvesting, the osteoblastic activity of the ipsilateral side of the sternum was lower than in the contralateral one.

**Figure 2:**
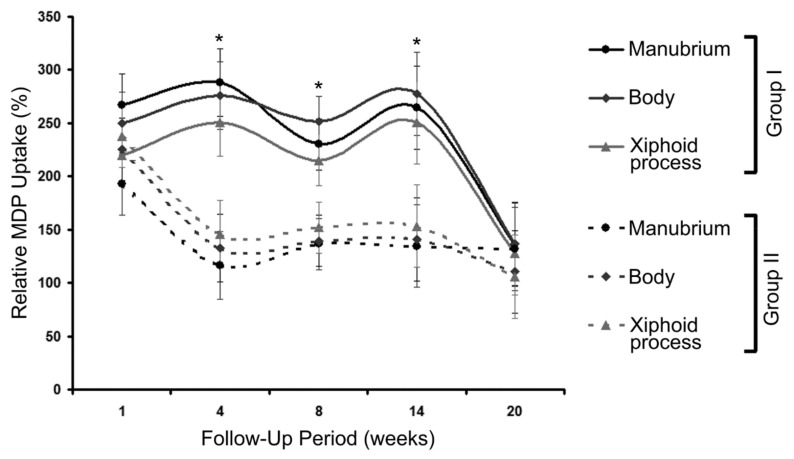
The changes in relative MDP uptake during 20 weeks of follow-up in groups I and II. * *p* < 0.05 using Dunn’s test.

The change in the MDP uptake was also studied during 20 weeks of follow-up in groups I and II. These data are presented in Figure 2. It demonstrates that during the first 14 weeks, the MDP uptake was approximately 1.5-fold higher in group I, but at the end of the follow-up, it was almost equal in both groups.

### 3.2. Sternal Perfusion after the ITA Harvesting, as Estimated Using 3PDS

The main disadvantage of SPECT is the inability to measure the ostial perfusion directly. The MDP uptake is measured 3 h after a radiopharmaceutical injection when its tissue redistribution takes place and thus cannot be interpreted as a perfusion indicator [12].

For a direct perfusion measurement, 3PDS with 99mTc MDP was performed 2 weeks after a CABG in groups I and II. In the sagittal plane, the ratio of the mean counts per pixel in the ROIs “B”, “D” and “F” indicated in Figure 1 were compared in groups I and II. For each ROI, the count curves were plotted for 2 min immediately after the radiopharmaceutical injection. This period included only the first (flow) phase.

**Figure 3:**
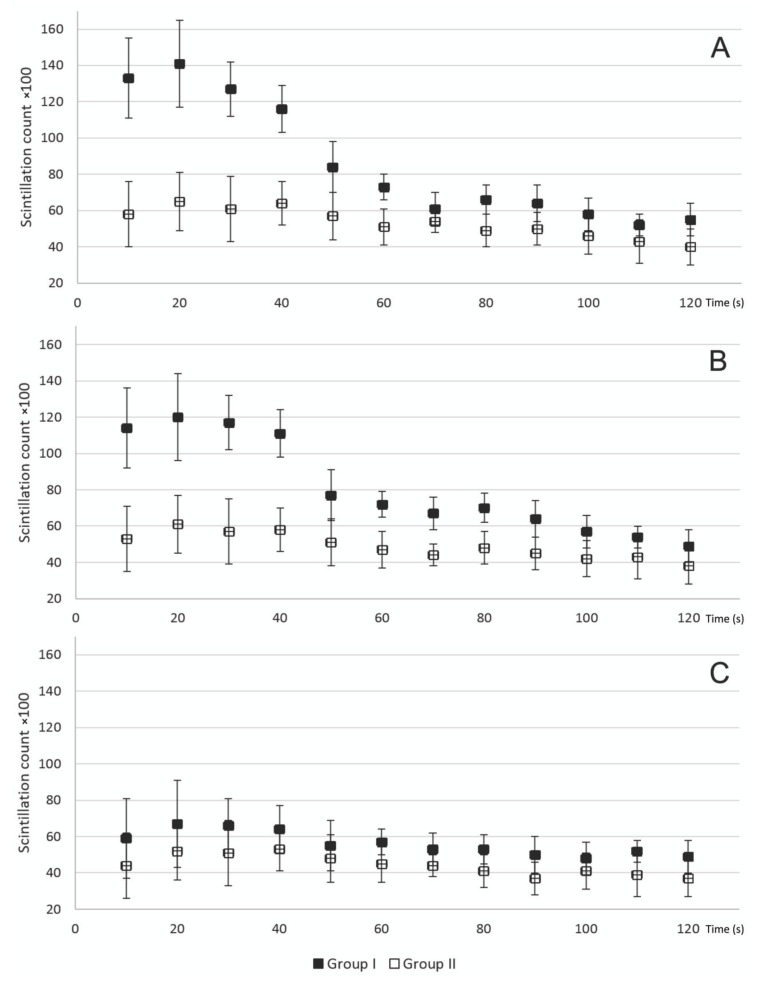
Comparison of the scintillation counts in the manubrium (**A**), body (**B**), and xiphoid process (**C**) ROIs in groups I and II. Note the large differences between the groups in scintillation counts in the body and especially in the manubrium region.

**Figure 4:**
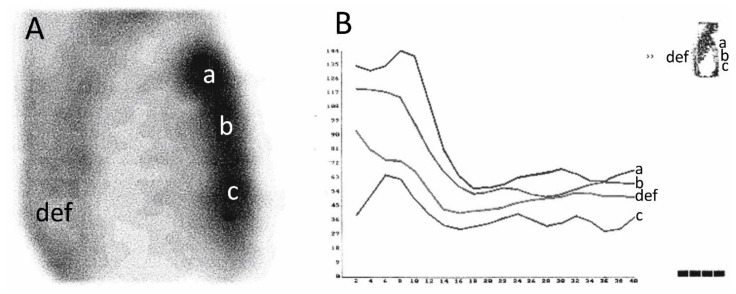
Single-photon emission computed tomography (SPECT) images of the thorax (**A**) and scintillation count curves (**B**) of patient after the ITA skeletonization. ROI: a—manubrium; b—body; c—xiphoid process; d, e, f—vertebral column. In the three-phasic dynamic scintigraphy (3PDS) during the flow phase, perfusion of the sternal manubrium and body was significantly more prominent due to their blood supply peculiarity and postoperative hyperemia. In the SPECT image, a significant MDP uptake was also found at the sternal manubrium and body.

**Figure 5:**
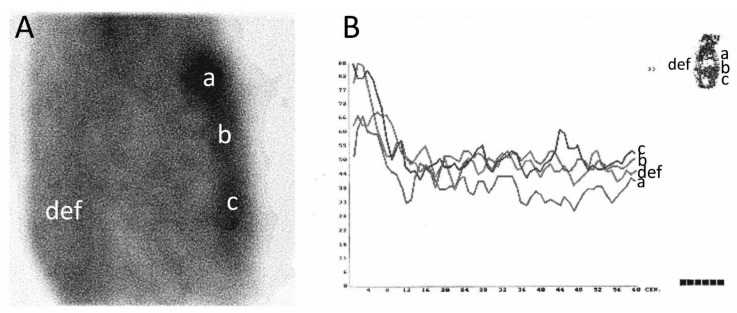
SPECT images of the thorax (**A**) and scintillation count curves (**B**) of patients after the pedicled ITA harvesting. ROI: a—manubrium; b—body; c—xiphoid process; d, e, f—vertebral column. In the 3PDS, the mean sternal MDP uptake was markedly lower than in the case described in Figure Figure 4. There was no significant difference in the scintillation count during the flow phase between all the ROIs. Meanwhile, the lowest count was observed in the manubrium, though in normal conditions, it must be the highest one because of a good vascularization by the ITA branches.

The comparison of the count curves is shown in Figure 3. Examples of the SPECT images of the thorax and 3PDS curves of patients after the ITA skeletonization and pedicled harvesting are shown in Figure 4 and Figure 5.

There was no statistically significant difference in the scintillation count in the xiphoid process ROI between groups I and II (*p* > 0.05), but in the manubrium and body ROIs, there was a significant difference (*p* < 0.05 using Dunn’s test). This means that the technique of ITA harvesting had an influence on the sternal perfusion, especially in the manubrium and body, where all three types of vessels supplying the sternum are mostly located.

The patient whose bone scan is shown in Figure 5 had osteomyelitis of the sternum a week later. These cases demonstrate that a week prior to a clinical manifestation of a sternal wound infection, the worsening of its blood supply, which is the main osteomyelitis risk factor, can be detected using 3PDS, and preventive therapy can be started earlier.

## 4. Discussion

In earlier studies, the MDP uptake by the sternum, as evaluated using SPECT, was incorrectly interpreted as a marker of its perfusion [13,14,15,16,17]. In a strict sense, this value represents only a bone osteoblastic activity, which only depends on sternal perfusion to a small degree. The perfusion itself can be obtained only using 3PDS. However, up to now, 3PDS has been ineffective for the evaluation of chest bones and the sternum because, after thoracic surgery, no specific SPECT patterns of sternal perfusion were revealed. This is explained by the fact that a pronounced soft tissue component of a scintillation count is superimposed onto the scintigrams of the chest bones [12]. Considering the main sternal blood supply origins, the heterogeneity of a scintillation count by different sternal segments made sense for our work.

Furthermore, in previous studies, the absolute scintillation count in the right and left halves of the sternum were evaluated. However, after the sternotomy, the absolute value of MDP uptake increases significantly in a way that can affect the results of studies. We undertook a different approach, which involved studying the MDP uptake using different segments of the sternum in relation to the vertebral column, the bone tissue of which is intact and, consequently, uptakes phosphates with physiological intensity. As with previous studies, there were no control groups of patients who did not undergo ITA harvesting.

As a result, we found that the highest sternal MDP uptake was in the group of patients with the skeletonized ITA. It was even higher than in the group without the ITA harvesting, probably because of compensatory hyperemia of a contralateral side of the sternum. This can happen because a clipping of a sternal perforation and intercostal branches of the ITA leads to an increase in sternal blood supply due to exclusion of the ITA, which accepts blood inflow from posterior intercostal arteries.

The lowest scintillation count was observed in the group with the pedicled ITA harvesting. The patients in all the groups were comparable in all factors influencing the sternal MDP uptake except for the ITA harvesting method. Therefore, the essential factor regarding postoperative sternal wound complications in the absence of fractures was the sternal blood supply worsening concomitant to the pedicled ITA harvesting. The lowest MDP uptake in the group of the pedicled ITA harvesting was observed mainly in a proximal part of the sternum. This confirmed the involvement of the internal thoracic artery branches supplying the sternum.

MDP has an equal affinity to both the organic bone matrix (osteoblasts, multinuclear phagocytes) and the hydroxyapatite crystal. Therefore, its uptake can increase due to inflammation (adsorption of phagocytes on a cellular membrane) or osteogenesis activity (intracellular and intercellular accumulation) [18]. We considered that the MDP accumulation in the sternum was higher than in the vertebral column because of the sternotomy, posttraumatic bone remodeling, and inflammation concomitant to surgery.

During the first 14 weeks of follow-up, the MDP uptake increased significantly (by more than 150%) in group I, but by the 20th week, these values were comparable in both groups. This can occur due to neoangiogenesis and collateral branch formation.

All the abovementioned facts confirmed that the ITA skeletonization allowed for an increase in the length and mobility of the ITA and did not worsen the postoperative sternum reparation. Similar data were obtained by other authors who used only SPECT in their work [19,20]. Recently, Kamiya et al., demonstrated that the damage of the tissue microcirculation in the middle and lower retrosternal area is significantly less after internal thoracic artery skeletonization compared with the pedicled internal thoracic artery harvesting technique [21]. These results, together with ours, prove that ITA skeletonization prevents the development of sternal ischemia and wound infections after a CABG.

The main limitation of our study was that the unilateral and bilateral ITA harvesting was not compared. Several studies have been performed to investigate the clinical safety of bilateral ITA harvesting and its effect on the risk of deep sternal infection [22,23,24,25]. However, we found no works, especially randomized, evaluating the effect of bilateral ITA harvesting on sternal perfusion.

## 5. Conclusions

The main factor that improved the sternal perfusion after a CABG was the preservation of branches supplying the sternum using the skeletonization technique of ITA harvesting.

## Figures and Tables

**Table 1:** Clinical characteristics of the studied patients.

Parameter	Group I (*n* = 18)	Group II (*n* = 18)	Group III (*n* = 21)	*P*_I–II–III_	*P*_I–II_
Mean age (years)	57.2 (53.1–63.4)	58.6 (53.2–64.7)	55.8 (51–61.3)	0.307	0.644
Male gender	18 (100%)	18 (100%)	21 (100%)	ns	ns
Myocardial infarction in anamnesis	9 (50%)	7 (39%)	0 (0%)	–	0.737
NYHA class	2.1 ± 0.4	2.2 ± 0.3	2.3 ± 0.4	0.097	0,298
Angina CCS class	2.4 ± 0.3	2.3 ± 0.2	0	–	0.516
Diabetes mellitus	0 (0%)	0 (0%)	0 (0%)	ns	ns
Number of coronary arteries with hemodynamically significant stenoses	1.8 ± 0.3	1.6 ± 0.3	0	–	0.793
Number of coronary grafts	1.8 ± 0.3	1.6 ± 0.3	0	–	0.629

Comment. NYHA—New-York Heart Association; CCS—Canadian Cardiovascular Society.

**Table 2:** Comparative MDP uptake by different segments of the sternum.

ROI	Relative MDP Uptake (%)			*p*			
	Group I (*n* = 18)	Group II (*n* = 18)	Group III (*n* = 21)	*P*_I–II–III_	*P*_I–II_	*P*_II–III_	*P*_I–III_
Manubrium	252.9 (199.3–288.8)	113.4 (104.9–171.5)	161.9 (158–172.4)	0.0017	<0.05	<0.05	<0.05
Body	236.1 (187.3–304.0)	119.1 (110.6–161.1)	132.2 (125.3–154.0)	0.0002	<0.05	<0.05	<0.05
Xiphoid process	207.7 (174.2–265.4)	128.5 (119.7–144.6)	142.1 (119.1–151.0)	0.0009	<0.05	<0.05	<0.05
Left to right side ratio	110.0 (106.3–119.8)	82.4 (76.1–92.3)	112.0 (104.3–139.0)	0.017	<0.05	<0.05	>0.05
Vertebral column	100.0	100.0	100.0	–	–	–	–
